# Effect of *Helicobacter pylori* eradication on reflux esophagitis and GERD symptoms after endoscopic resection of gastric neoplasm: a single-center prospective study

**DOI:** 10.1186/s12876-020-01276-1

**Published:** 2020-04-21

**Authors:** Hee Kyong Na, Jeong Hoon Lee, Se Jeong Park, Hee Jung Park, Sun Ok Kim, Ji Yong Ahn, Do Hoon Kim, Kee Wook Jung, Kee Don Choi, Ho June Song, Gin Hyug Lee, Hwoon-Yong Jung

**Affiliations:** 1 Department of Gastroenterology, University of Ulsan College of Medicine, Asan Medical Center, 88, Olympic-ro 43-gil, Songpa-gu, Seoul, 05505 South Korea; 2 Department of Clinical Epidemiology and Biostatistics, University of Ulsan College of Medicine, Asan Medical Center, 88, Olympic-ro 43-gil, Songpa-gu, Seoul, 05505 South Korea

**Keywords:** *Helicobacter pylori*, Eradication, Reflux esophagitis, Gastroesophageal reflux

## Abstract

**Background:**

The association between *Helicobacter pylori* and reflux esophagitis (RE) remains controversial. This study aimed to prospectively evaluate the effect of *H. pylori* eradication on RE and gastroesophageal reflux (GERD) symptoms in *H. pylori*-positive patients who underwent endoscopic resection of gastric neoplasm.

**Methods:**

Of the 244 patients enrolled in this study, 173 *H. pylori*-positive patients underwent follow-up at least once. We evaluated the prevalence of RE and GERD symptoms in these patients following *H. pylori* eradication.

**Results:**

There were 75.7% (131/173), 78.6% (125/159), and 78.9% (105/133) subjects who were successfully eradicated after 6, 12, and 18–24 months, respectively. During the 2-year follow-up period, the eradication of *H. pylori* did not increase the incidence of RE (OR 0.93; 95% CI, 0.49–1.77, *p* = 0.828). *H. pylori* status was also not associated with the development of GERD symptoms (OR 1.12; 95% CI, 0.47–2.95, *p* = 0.721). In the univariate analysis for RE, present smoking history (OR 4.79; 95% CI 1.98–11.60, *p* = 0.001), present alcohol consumption history (OR 2.18; 95% CI 1.03–4.63, *p* = 0.041), and diabetes mellitus (OR 2.44; 95% CI 1.02–5.86, *p* = 0.045) were found to be associated with RE. Multivariate analysis showed that present smoking history (OR 4.54; 95% CI 1.84–11.02, *p* = 0.001) was a significant risk factor for RE.

**Conclusions:**

*H. pylori* eradication did not increase the incidence of RE or GERD symptoms in patients who underwent endoscopic resection of gastric neoplasm.

## Background

*Helicobacter pylori* plays a key role in the pathogenesis of various diseases such as peptic ulcers, gastric mucosa-associated tissue lymphoma, and intestinal-type gastric cancer. Since Schutze et al. first mentioned in 1995 that reflux esophagitis (RE) was developed after *H. pylori* eradication [1], numerous studies on the association of *H. pylori* with RE or gastroesophageal reflux (GERD) symptoms have been reported. Reports have shown that *H. pylori* infection is negatively associated with erosive esophagitis and Barrett’s esophagus but not with GERD symptoms [2–5].

Based on the study showing that *H. pylori* eradication significantly lowers the incidence of metachronous lesion in patients who underwent endoscopic resection of early gastric cancer, we evaluated the effect of *H. pylori* eradication on RE and GERD symptoms in a large cohort of patients who underwent endoscopic resection of gastric epithelial neoplasm and had *H. pylori* infection. These patients, who are less likely to lose follow-up, are relatively homogenous. The aim of our study was to evaluate the effect of *H. pylori* eradication on RE and GERD symptoms in a large cohort of patients who underwent endoscopic resection of gastric epithelial neoplasm and had *H. pylori* infection.

## Methods

### Patients

We conducted a single-center prospective study. Enrolled patients were aged 20–70 years who underwent endoscopic resection including endoscopic mucosal resection and endoscopic submucosal dissection of gastric neoplasm at Asan Medical Center, Seoul, Korea, from September 2012 to March 2014. Patients were excluded on the basis of the following criteria: (1) *H. pylori*-negative, (2) presence of upper gastrointestinal bleeding, (3) presence of esophageal or gastric varices, (4) presence of esophageal or pyloric stenosis, (5) previous history of gastrectomy, (6) history of *H. pylori* eradication, (7) presence of tumor at the gastroesophageal junction or pylorus resulting in outlet obstruction, (8) past or present history of taking H2 receptor blocker or proton pump inhibitor (PPI) within at least 1 month, (9) alcohol or drug abuse, and (10) being pregnant. Written informed consent was obtained from all participants. The study was conducted according to the guidelines of the Declaration of Helsinki and was approved by the Institutional Review of Board of Asan Medical Center (IRB number: 2014-2-10).

### Questionnaires

Patients who agreed to participate in the study answered questionnaires regarding GERD symptoms initially, at 6 months, and at 12 months after endoscopic resection. The questionnaire (Supplementary file) which we developed included questions regarding typical reflux symptoms; heartburn and acid regurgitation. If the patients replied yes to the questions, they were provided with further questions regarding the symptom severity that was categorized as mild, moderate, and severe. Body mass index (BMI) and status of alcohol consumption and smoking were recorded.

### Endoscopic examinations

The endoscopist was blinded to the result of the questionnaires. The presence or absence of RE was evaluated initially when the patient underwent endoscopic resection. The severity of RE on EGD was graded in accordance with the Los Angeles classification system [6]. Minor changes in the squamocolumnar junction, such as blurring or hyperemic changes were not considered as RE. Gastroesophageal junction extending more than 2 cm from the diaphragmatic impingement determined hiatal hernia [7]. Gastric mucosal atrophy was evaluated and the atrophic type was divided into closed and open types according to endoscopicatrophic border. Closed-type atrophic gastritis (antral predominant gastritis) was diagnosed when the atrophic border remained on the lesser curvature of the corpus, whereas open-type atrophic gastritis (pangastritis or corpus predominant gastritis) was diagnosed when the atrophic border no longer existed on the lesser curvature, but extended along the anterior and posterior walls of the stomach [8].

The serum levels of pepsinogens I (PGI) and II (PGII) were evaluated using Pepsinogen I and Pepsinogen II EIA TEST kits (BiohitOyj, Helsinki, Finland). When PGI levels were < 25 μg/L and PGI to PGII ratio was < 3, serological atrophy was defined to be present [9, 10].

### Eradication of *H. pylori* and follow-up

*H. pylori* infection was determined using a rapid urease test, a ^13^Curea breath test, and histology. For histological confirmation, two biopsy specimens were obtained from the greater curvature of the body, and the antrum and the specimens were examined after Giemsa staining. At least one positive result of the above tests was assumed as present *H. pylori* infection. If the patients showed positive results for *H. pylori*, eradication therapy was provided. As a first-line therapy, full-dose PPI, 500-mg clarithromycin, and 1000-mg amoxicillin, twice for 7–14 days, were prescribed. ^13^Curea breath test or rapid urease test was performed for confirming the success of eradication therapy after at least 4 weeks from the completion of eradication therapy. In the event that the patient failed triple therapy, bismuth-based quadruple therapy was initiated that consisted of full-dose PPI b.i.d., 500-mg metronidazole t.i.d., 500-mg tetracycline q.i.d., and 120-mg bismuth t.i.d. for 7–14 days.

Full-dose PPI was usually prescribed for 4 weeks after endoscopic resection. Follow-up EGD was performed after 6 months for 2 years, and we evaluated the presence of RE and *H. pylori* status using the rapid urease test.

### Statistical analysis

Continuous variables were reported as means ± standard deviations. Depending on the distribution, the two-sample t-test or the Mann–Whitney U test was used for comparing continuous variables. All *P*-values were two-sided, and P-values of < 0.05 were considered statistically significant. For analyzing the probability of RE and GERD symptoms, generalized estimating equation with logistic regression was used. Risk factors for RE and GERD symptoms were assessed using univariate and multivariate analyses with logistic regression analysis. All statistical analyses were performed using the statistical package for the social sciences software (SPSS version 21.0, Chicago, IL, USA).

## Results

### Study population

Of the 244 patients who were enrolled during the study period, 25 patients underwent surgery, 18 patients were lost to follow-up, and 28 patients who initially showed negative *H. pylori* results were excluded. Therefore, we analyzed the data of 173 patients who initially showed *H. pylori*-positive results. Baseline characteristics of this study population are presented in Table 1. The percentage of males was found to be 76.9% and the mean age was 58 years. On final pathology of resected specimen, low-grade dysplasia was diagnosed in 61 cases (35.3%), high-grade dysplasia in 18 cases (10.4%), early gastric cancer in 92 cases (53.2%), neuroendocrine tumor in one case (0.6%), and gastritis in one case (0.6%; previous biopsy specimen was indicated as high-grade dysplasia, but final pathology did not show dysplastic lesions). Hiatal hernia was observed in 25/173 cases (14.5%). The prevalence of initial RE was 1.7% (3/173) and all the patients showed mild esophagitis, LA-A. The prevalence of initial GERD such as heart burn and acid regurgitation was 26.6% (46/173).

**Table 1:** Baseline characteristics of the study population

Variables	Total (*n* = 173)
Number	173
Male/female	133/40
Age	58.0 ± 8.0
Body mass index	
< 25	103 (59.5)
25–30	63 (36.4)
30–35	6 (3.5)
> 35	1 (0.6)
Past history	
HTN	47 (27.2)
DM	25 (14.5)
Hyperlipidemia	17 (9.8)
Final pathology of resected specimen	
Low-grade dysplasia	61 (35.3)
High-grade dysplasia	18 (10.4)
Mucosal cancer	89 (51.4)
Submucosal cancer	3 (1.7)
Others	2 (1.2)
Smoking	
Current	34 (19.7)
Past	58 (33.5)
None	81 (46.8)
Alcohol consumption	
Current	87 (50.3)
Past	19 (11.0)
None	67 (38.7)
Hiatal hernia	25 (14.5)
Atrophic gastritis	
None	1 (0.6)
Open type	132 (76.3)
Closed type	40 (23.1)
Fasting glucose, mg/dL	116.0 ± 41.5
Total cholesterol, mg/dL	182.7 ± 33.2
Pepsinogen level	
Pepsinogen I, ng/mL	97.1 ± 68.4
Pepsinogen II, ng/mL	18.4 ± 10.7
Pepsinogen I/II ratio	5.8 ± 3.2

Values are presented with numbers (%) and mean ± standard deviation

At 6, 12, and 18–24 months after endoscopic resection, 173, 159, and 133 patients, respectively, were followed up. During the study period, 32 patients failed the first line eradication therapy, two patients refused for further eradication, and 30 patients underwent second line regimens. Among four patients who showed sustained positive result after the second line therapy, three underwent third line regimen (sequential therapy).

### Prevalence of RE and GERD symptoms

**Fig. 1:**
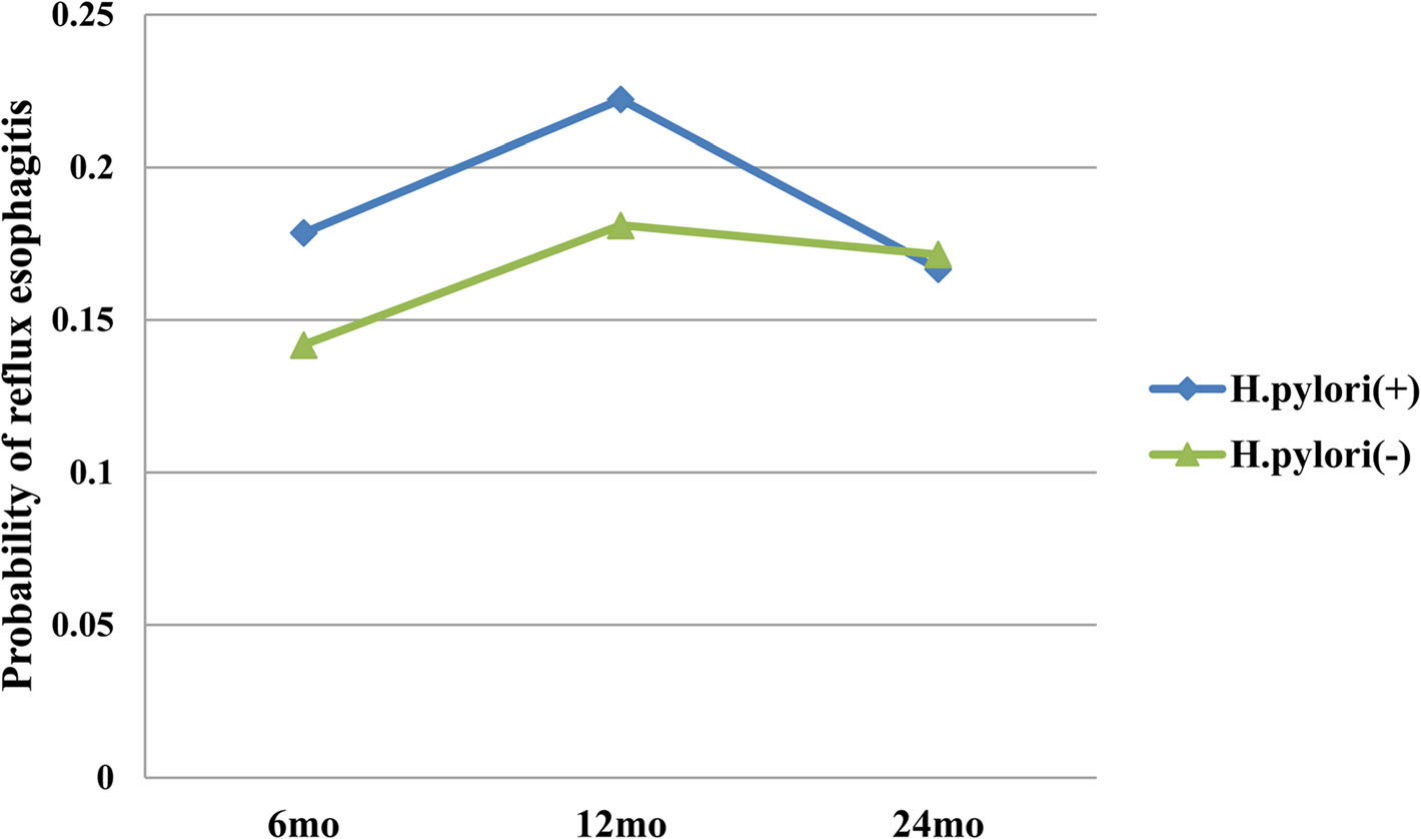
Probability of reflux esophagitis (*P* = 0.95)

**Fig. 2:**
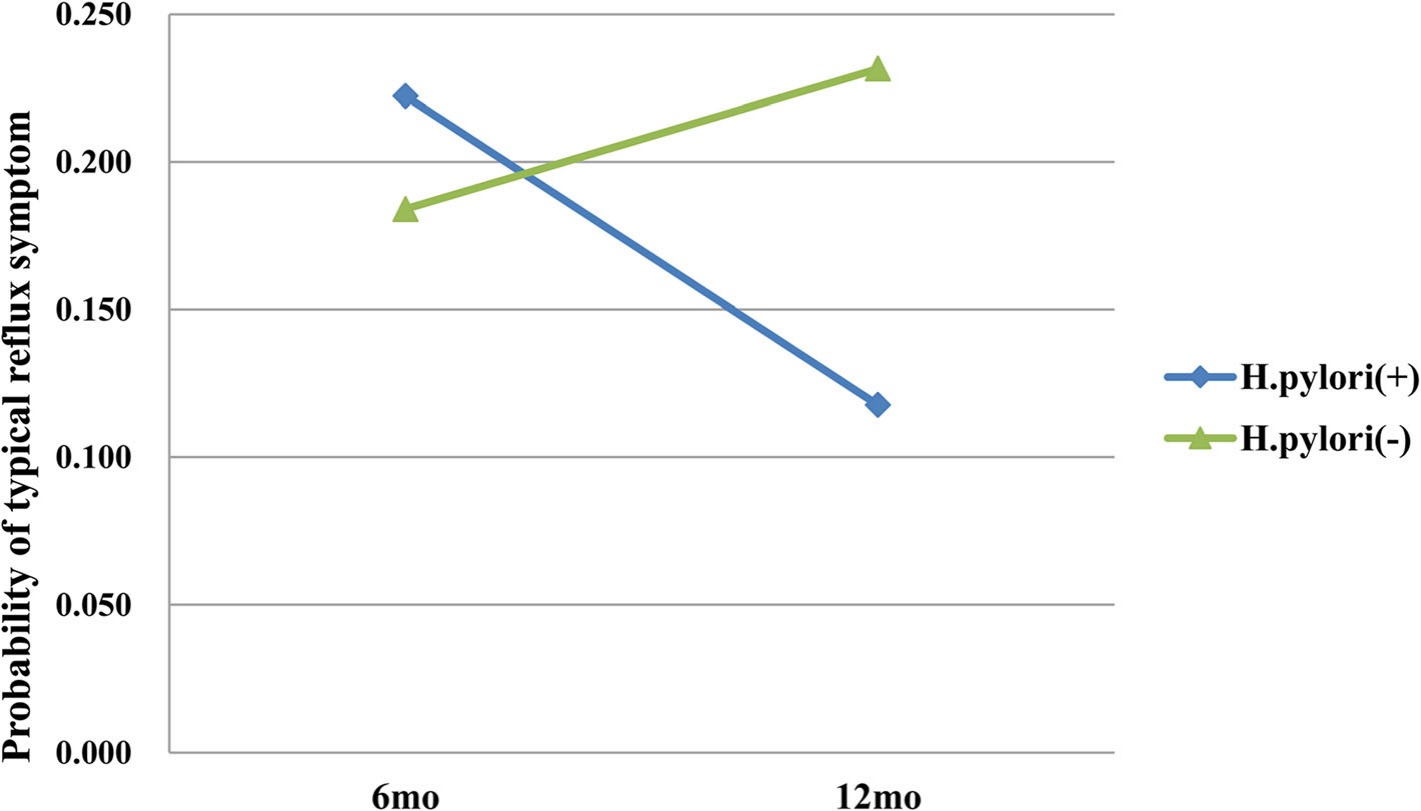
Probability of reflux symptoms (*P* = 0.18)

There were 75.7% (131/173), 78.6% (125/159), and 78.9% (105/133) subjects who were successfully eradicated after 6, 12, and 18–24 months, respectively. The prevalence of RE and GERD symptoms during follow-up period is presented in Table 2. The prevalence of RE and GERD symptoms between *H. pylori*-positive and *H. pylori*-negative patients is not significantly different at 6, 12, and 18–24 months after endoscopic resection. The probabilities of RE that failed to show a difference in both groups were as follows: 0.18 in *H. pylori*-positive vs. 0.14 in *H. pylori*-negative at 6 months, 0.22 vs. 0.18 at 12 months, and 0.17 vs. 0.17 at 18–24 months after endoscopic resection, respectively (*P* = 0.95, Fig. 1). The probabilities of reflux symptoms that failed to show a difference in both groups were as follows: 0.22 in *H. pylori*-positive vs. 0.18 in *H. pylori*-negative at 6 months and 0.12 vs. 0.23 at 12 months, respectively (*P* = 0.18, Fig. 2).

**Table 2:** Prevalence of reflux esophagitis and typical reflux symptoms after endoscopic resection

Follow-up		*H. pylori*-positive	*H. pylori*-negative	*P-*value
6 months	Reflux esophagitis	5/28 (17.9)	18/127 (14.2)	0.569
	Reflux symptoms	6/27 (22.2)	24/129 (18.6)	0.664
12 months	Reflux esophagitis	4/18 (22.2)	21/116 (18.1)	0.745
	Reflux symptoms	2/17 (11.8)	26/111 (23.4))	0.360
18–24 months	Reflux esophagitis	2/12 (16.7)	18/10 (17.1)	1.000

### Factors associated with RE and GERD symptoms

Univariate analysis for risk factors associated with RE is shown in Tables 3 and 4. Younger age (OR 3.71; 95% CI, *P* < 0.012), present smoking status, alcohol consumption, and diabetes mellitus were associated with RE. In multivariate analysis, present smoking was noted to be the only significant risk factor for RE (Table 4).

**Table 3:** Univariate analysis for risk factors of reflux esophagitis

Risk factor		Number	Event	Odds ratio	95% CI		*P*-value
Sex	Male	133	40	2.028	0.828	4.967	0.122
	Female	40	7	1			
Age				0.956	0.917	0.996	0.032
Body mass index	< 25	103	25	1			0.468
	25–30	63	19	1.347	0.668	2.718	0.405
	> 30	7	3	2.340	0.490	11.599	0.286
Smoking	Present	34	17	4.786	1.975	11.599	0.001
	Past	58	16	1.823	0.807	4.116	0.148
	Non	81	14	1			0.002
Alcohol consumption	Present	87	30	2.186	1.033	4.627	0.041
	Past	19	4	1.108	0.315	3.898	0.873
	Non	67	13	1			0.090
Hiatal hernia	yes	25	7	1			
	no	148	40	1.050	0.408	2.703	0.919
Hypertension	no	126	36	1			
	yes	47	11	0.764	0.351	1.663	0.497
Hypercholesterolemia	no	156	45	1			
	yes	17	2	0.329	0.072	1.497	0.150
Diabetes mellitus	no	148	36	1			
	yes	25	11	2.444	1.020	5.861	0.045
Totalcholesterol		173		1.007	0.997	1.017	0.188
Glucose		173		1.005	0.998	1.013	0.189

*CI* confidential interval

**Table 4:** Multivariate analysis for risk factor of reflux esophagitis

Risk factor		Odds ratio	95% CI		*P*-value
Smoking	Present	4.502	1.839	11.019	0.001
	Past	1.844	0.811	4.189	0.144
	Non	1			
Diabetes mellitus	no	1			
	yes	2.162	0.864	5.412	0.100

*CI* confidential interval

In univariate analysis for factors associated with reflux symptoms, *H. pylori* infection had a positive association with male sex (OR 3.71; 95% CI, 1.33–10.33, *P* < 0.012). Otherwise, risk factors such as BMI and the presence of hiatal hernia were not associated with reflux symptoms.

## Discussion

In the present study, we evaluated the probability of RE and reflux symptoms among patients with positive *H. pylori* who underwent endoscopic resection of gastric neoplasm. During the follow-up period, no significant difference was noted in the development for RE and reflux symptoms between *H. pylori*-positive and *H. pylori-negative* patients. We assumed that eradication therapy did not result in the development of RE or GERD symptoms.

It has been reported that *H. pylori* infection protects against the development of erosive esophagitis [11–14]. The first clinical trial suggested that the presence of *H. pylori* reduced the requirement of PPI [15]. In a study of patients with 244 duodenal ulcer, erosive esophagitis developed more frequently in patients treated with *H. pylori* eradication than that in *H. pylori*–positive patients [11]. However, more recent studies have suggested that *H. pylori* eradication does not have a clinical impact on RE. In a meta-analysis involving 11 randomized controlled trials, no difference in erosive esophagitis and reflux symptoms (reflux and heartburn) was noted between *H. pylori* eradication and persistent groups. In another meta-analysis including 10 trials, no statistically significant difference was found for endoscopic evidence of RE (OR 1.13; 95% CI 0.72–1.78, *p* = 0.59) or symptomatic GERD (OR 0.81, 95% CI 0.56–1.17, *p* = 0.27) between *H. pylori* treatment and the no-treatment groups [16]. A recent Japanese study that enrolled 8123 individuals for an annual checkup reported that the risk of RE in patients who underwent *H. pylori* eradication was even lower than those who were never infected [17].

GERD symptoms are relatively subjective and are not usually correlated with an endoscopic finding or a result from 24-h pH monitoring. Reports have demonstrated that *H. pylori* eradication does not lead to the development of GERD symptoms [5, 18]. In a post-hoc analysis of eight double-blind prospective studies, *H. pylori* eradication did not result in the development of new symptomatic GERD or worsening of symptoms in patients with preexisting GERD [19].

Multiple pathophysiological factors influence the development and course of RE, and gastric acid secretion is known to be a key factor in the pathophysiology of the disease. The possible underlying mechanism of *H. pylori* infection is associated with gastric acid output. In the early stage of the study, we postulated that our study population who primarily had severe atrophic gastritis and acid hyposecretion state would develop RE or GERD symptom after *H. pylori* eradication. Actually, many patients (76.3%) showed open-type gastric atrophy and all patients except one showed atrophic changes. However, RE or GERD symptoms were not different between the two groups. In a prospective double-blind study measuring 24-h esophageal pH for established GERD patients, esophageal acid exposures were not different between *H. pylori*-positive and *H. pylori*-negative patients [20]. Another study evaluating 112 patients with GERD symptoms also showed that *H. pylori* infection did not affect esophageal acid exposure [21]. In patients with GERD without *H. pylori* infection, pathophysiological studies show that transient lower esophageal sphincter relaxations are the predominant mechanism of reflux [22].

In our study, smoking was the only risk factor for the development of RE. A recent study evaluated the risk factors for RE after the eradication of *H. pylori*; male sex, BMI ≥ 25 kg/m^2^, the use of calcium channel blocker, and hiatal hernia were associated with the development of RE [23]. In another study evaluating the long-term effect of *H. pylori* eradication on the prevalence of RE, male sex, higher BMI, habitual alcohol consumption, habitual smoking, larger diaphragmatic hiatal size, and milder gastric mucosal atrophy were noted to be risk factors for RE [17].

Our study has several limitations. First, the prevalence of gastric atrophy was relatively higher in our study population and patients who have gastric neoplasm are not representative of the screened population. Second, the study was performed in a single center in Korea and ethnic variation could not be considered. Third, we did not perform functional study of gastroesophageal junction, which could have provided additional physiological evidence. Fourth, the questionnaire for GERD symptom evaluation was not validated. Despite these limitations, this is a prospective study that analyzes RE and GERD symptoms after *H. pylori* eradication among patients who underwent endoscopic resection of gastric neoplasm.

## Conclusions

*H. pylori* eradication did not increase the incidence of endoscopic RE or typical GERD symptoms in patients who underwent endoscopic resection of gastric neoplasm.

## Supplementary information

**Additional file 1.**

## Data Availability

The datasets used and/or analyzed during the present study are available from the corresponding author on reasonable request.

## Abbreviations

GERD: Gastroesophageal reflux disease
RE: Reflux esophagitis
*H.pylori*: *Helicobacter pylori*
OR: Odd ratio
CI: Confidential interval
BMI: Body mass index
PPI: Proton pump inhibitor
PG: Pepsinogen
