# Supplementary material for: Effect of Helicobacter pylori eradication on reflux esophagitis and GERD symptoms after endoscopic resection of gastric neoplasm: a single-center prospective study
Source: BMC Gastroenterol. 2020 Apr 21;20:123. doi: 10.1186/s12876-020-01276-1 (PMC7175488; doi:10.1186/s12876-020-01276-1)
Supplement: Supplementary file 1 — Additional file 1. [file 12876_2020_1276_MOESM1_ESM.docx]

| **GERD Symptom Questionnaires** |
| --- |

Name : Age/Gender : Date:

(1) Please check the following questions.

| Question | | answer | |
| --- | --- | --- | --- |
| 1 | Do you get heartburn? | yes | no |
| 2 | Do you get bitter liquid (acid) or foods material comping up into your throat? | yes | no |
| 3 | Do you have epigastric or chest pain? | yes | no |
| 4 | Do you have chronic cough? | yes | no |
| 5 | Do you have a hoarse voice? | yes | no |
| 6 | Do you feel foreign body sensation in your throat? | yes | no |
| 7 | Do you feel that you are wheezing? | yes | no |

(2) If you answer yes for the questions, check the appropriate response below.

| 번호 | 질문 | Never | Mild | Moderate | Severe |
| --- | --- | --- | --- | --- | --- |
| 1 | Heartburn |  |  |  |  |
| 2 | Bitter liquid (acid) or foods material comping up into your throat |  |  |  |  |
| 3 | Epigastric or chest pain |  |  |  |  |
| 4 | Heartburn when lying down |  |  |  |  |
| 5 | Bitter liquid or acid in throat |  |  |  |  |

3) Have you had any of the following difficulties during the past 2 weeks due to gastrointestinal symptoms?

| 번호 | 질문 | Never | Occasionally | Sometimes | Often | Always |
| --- | --- | --- | --- | --- | --- | --- |
| 1 | I avoided daily activities. |  |  |  |  |  |
| 2 | I did not want to participate in social activities. |  |  |  |  |  |
| 3 | I had concentration problem. |  |  |  |  |  |
| 4 | I avoided physical activities or exercise. |  |  |  |  |  |
| 5 | I worried what I can and cannot eat. |  |  |  |  |  |
| 6 | I avoided certain kinds of food. |  |  |  |  |  |
| 7 | I could not sleep or I woke up in the middle of sleeping. |  |  |  |  |  |
| 8 | I felt bad and irritable. |  |  |  |  |  |
| 9 | I was anxious and depressed. |  |  |  |  |  |
| 10 | I felt lethargic. |  |  |  |  |  |
